# Expression of *E-CADHERIN* and *miR-200b* in Different Forms of Endometriosis

**DOI:** 10.3390/biomedicines13020524

**Published:** 2025-02-19

**Authors:** Konstantinos Ntzeros, Charalampos Voros, Despoina Mavrogianni, Nikolaos Kathopoulis, Konstantinos Kypriotis, Antonia Varthaliti, Menelaos Darlas, Athanasios Douligeris, Athanasios Protopapas

**Affiliations:** 1 Experimental Laboratory, 1st Department of Obstetrics and Gynecology, Medical School, National and Kapodistrian University of Athens, 11528 Athens, Greece; kntzeros@gmail.com (K.N.); depy.mavrogianni@yahoo.com (D.M.); antonia.varthaliti@hotmail.com (A.V.); mdarlas2110@gmail.com (M.D.); 2 Endoscopic Surgery Unit, 1st Department of Obstetrics and Gynecology, Medical School, National and Kapodistrian University of Athens, 11528 Athens, Greece; nickatho@gmail.com (N.K.); kypriotiskonstantinos@yahoo.it (K.K.); thanosdouligeris92@gmail.com (A.D.);

**Keywords:** endometriosis, *E-CADHERIN*, *miR-200b*, gynecology, ovarian endometriosis

## Abstract

**Background/Objectives:** Epithelial–Mesenchymal Transition (EMT) is the process by which epithelial cells acquire mesenchymal properties, which helps endometriotic cells migrate and invade. This study looks at the expression of *E-CADHERIN*, a critical epithelial marker, and *miR-200b*, an EMT regulator, in several types of endometriosis, including endometriomas and deep infiltrating endometriotic (DIE) nodules. **Methods:** We examined 19 individuals with endometriosis (9 with just endometriotic cysts and 10 with both DIE and endometriotic cysts) and 8 controls with benign gynecological abnormalities. Tissue samples were taken during laparoscopic surgery, and *E-CADHERIN* and *miR-200b* expression were measured using Real-Time PCR, with *G6PD* and *U6* as controls. **Results:** *E-CADHERIN* expression was maintained in the eutopic endometrium of both ovarian and DIE types, but it was considerably reduced in endometriotic cysts, indicating heightened mesenchymal features. *miR-200b* was downregulated in the eutopic endometrium of ovarian endometriosis but upregulated in DIE. Endometriotic cysts in both groups had greater *miR-200b* expression than their corresponding eutopic endometrium. *E-CADHERIN* and *miR-200b* expression in DIE lesions was similar to that found in matched eutopic endometrium. **Conclusions:** The regulation of *E-CADHERIN* and *miR-200b* varies across ovarian and DIE lesions. The *miR-200b-ZEB1* feedback loop is increased in DIE eutopic endometrium but downregulated in ovarian endometriosis. *E-CADHERIN* downregulation in endometriotic cysts indicates heightened mesenchymal dynamics, whereas DIE nodules have gene expression patterns similar to eutopic endometrium. These findings emphasize the distinct regulatory processes that govern endometriotic lesions.

## 1. Introduction

Endometriosis is a benign estrogen-dependent inflammatory condition affecting women of reproductive age, and it is mainly associated with pelvic pain and infertility. It is defined as the presence of endometrial glands and stroma outside the endometrial cavity [1]. Endometriosis is estimated to affect up to 10% of women of reproductive age [1]. Several theories have been proposed, and molecular pathways have been associated with this entity in order to explain the complex underlying pathogenetic mechanisms of endometriosis development. So far, none of the theories fully explain this heterogeneous clinical disease or the different forms of endometriotic lesions. There is a growing belief that the problem pre-exists in the eutopic endometrium with inherited genetic/epigenetic defects, which create a predisposition for endometriosis, while the ectopic environment is a major factor that drives the development of the lesion [2]. Epithelial–Mesenchymal Transition (EMT) is a multi-stage cellular process where an epithelial phenotype acquires mesenchymal characteristics. In endometriosis, EMT is believed to enhance the migratory and invasive abilities of the endometriotic cell [3]. The most important effect of EMT in an epithelial cell is the downregulation of *E-CADHERIN* expression, which is regulated by several mesenchymal transcription factors such as *SNAIL*, *SLUG*, *TWIST*, and *ZEB1* [3,4]. In a previous article, we investigated the expression profile of *ZEB1* in different forms of endometriosis, finding different expression levels between eutopic endometrium and endometriotic lesions [5]. In this article, we focused on *E-CADHERIN*, which is a major marker of epithelial phenotype, and *miR-200b* that regulates *ZEB1* expression by a double-negative feedback loop [6]. The aim of this study was to evaluate the potential differential expression of *E-CADHERIN* and *miR-200b* in deeply infiltrating endometriotic (DIE) lesions, in ovarian endometriomas, and in the eutopic endometrium of women with endometriosis, in order to ascertain whether the EMT pathway *miR-200b*-*ZEB1*-*E-CADHERIN* is differentially regulated in different forms of endometriosis. Our results indicate that the EMT pathway miR-200b-ZEB1-E-CADHERIN is differentially regulated in different forms of endometriosis. This variation in gene expression supports the idea that deep infiltrating endometriosis (DIE) and ovarian endometriosis (EMAs-only) are caused by distinct molecular pathogenetic pathways rather than the same diseased entity. The different amounts of E-CADHERIN downregulation, ZEB1 activation, and miR-200b expression in these lesions show that EMT is regulated by local microenvironmental factors such as hormonal impacts and oxygen availability. These findings underscore the importance of a tiered approach to endometriosis classification and treatment that takes into account genetic variations across lesion types. Finding dissimilar differential gene expression between different forms of endometriosis will support the idea that they are established through different molecular pathogenetic mechanisms and, as a consequence, should be considered as different pathological entities.

## 2. Material and Methods

### 2.1. Sample Collection

We obtained eutopic endometrium, endometriomas, and DIE nodule tissue samples from 19 endometriosis patients who were treated laparoscopically in the 1st Department of Obstetrics and Gynecology at ’Alexandra’ General Hospital, National and Kapodistrian University of Athens. The existence of endometriosis was established both surgically and histopathologically. Additionally, eutopic endometrial samples were taken from 8 individuals with benign gynecological abnormalities but no endometriosis.

Women with endometriosis were separated into two groups based on the type of endometriosis that afflicted them. The first research group consisted of 9 women with endometriotic cysts but no DIE (endometriomas-only group, EMAs-only), whereas the second group consisted of 10 women with DIE who had also developed endometriotic cysts. Women without endometriosis were selected as the control group, which included four patients who were treated laparoscopically for leiomyomas, and four with adnexal cysts, other than endometriomas. The procedure of tissue sample collection, the demographic information of the patients as well as the description of the endometriotic lesion are reported in our previous article [5].

### 2.2. RNA Isolation, Reverse Transcription and Quantitative Real-Time PCR

The samples were treated with Monarch Total RNA Miniprep Kit (#T2010S; New England BioLabs Inc., Ipswich, MA, USA) for total RNA extraction, LunaScript RT SuperMix Kit (#E3010L; New England BioLabs Inc.) for *E-CADHERIN* gene, and Mir-X miRNA First-Strand Synthesis Kit for *miR-200b* (638315; Takara Bio USA, Inc., San Jose, CA, USA) for cDNA synthesis. The *G6PD* gene was employed as an internal control to standardize the expression levels of *E-CADHERIN* and *U6* for *miR-200b*. The primers for RT-PCR were for the *E-CADHERIN* gene (*CDH1*): Forward 5′-GCCTCCTGAAAAGAGAGTGGAAG-3′, Reverse 5′-TGGCAGTGTCTCTCCAAATCCG-3′; G6PD gene: Forward 5′-TGGACCTGACCTACGGCAACAGATA-3′, Reverse: 5′-GCCCTCATACTG-GAAACCC-3′; miR-200b gene: Forward 5′-CTTACTGGGCAGCATTG-3′, Reverse 5′-GAACATGTCTGCGTATCTC-3′. All primers were acquired from Eurofins Genomics GmBH in Germany. The 2^−ΔΔCT^ technique [7] was used to calculate the relative mRNA expression levels. The expression levels of a gene were investigated in lesions and compared to their paired eutopic endometrium, the expression difference was expressed as fold change according to the 2^−ΔΔCT^ method. We employed the same method to compare the eutopic endometrium of a research group to that of a control group. We utilized the dCp parameter to assess expression levels between lesions (endometriomas versus DIE nodules or endometiomas in the EMAs-only vs. DIE groups). The 2^−ΔΔCT^ method’s dCp parameter normalizes the expression of a gene of interest to a housekeeping gene in the same sample (*G6PD* for *E-CADHERIN*, *U6* for *miR-200b*). As a result, the dCp represents the corrected Ct value of a gene of interest after removing the Ct value of a housekeeping gene. Using dCp values, we may compare various endometriotic samples without comparing them to their corresponding eutopic endometrium.

### 2.3. Statistical Analysis

The mean and range values of the quantitative data were calculated. The statistical differences in the groups were examined by one-sample *t*-test, two-sample *t*-test (independent samples or paired), and their non-parametric analogs such as the Wilcoxon Signed Rank test and Mann–Whitney U test. SPSS version 20 software was used for statistical analysis. The criterion of statistical significance was set at 5%.

### 2.4. Ethical Approval

The study protocol was approved by the Ethics Committee of ‘Alexandra’ General Hospital and a signed informed consent was obtained from each participant of the present study.

## 3. Results

### 3.1. Control Samples

To ensure that the lesions did not impact the expression of *E-CADHERIN* and *miR-200b* in the eutopic endometrium, we chose two populations with distinct benign gynecological lesions (leiomyomas and non-hormone dependent adnexal lesions) as a control group. The statistical difference between the two groups produced no significant findings for *E-CADHERIN* (*p* = 0.773) or *miR-200b* (*p* = 0.248). As a result, we grouped all of the samples together.

### 3.2. Gene Expression in Eutopic Endometrium

We looked at the expression levels of *E-CADHERIN* and *miR-200b* in the eutopic endometrium of women with endometriosis as a whole (EMAs-only and DIE group together, *n* = 19), without dividing them into subgroups based on the type of endometriosis that affected them, and compared them to the eutopic endometrium of women without endometriosis (*n* = 8). There was no statistically significant difference between women with and without endometriosis in terms of *E-CADHERIN* (*p* = 0.982) or *miR-200b* (*p* = 0.836). We performed the same comparison for *ZEB1* gene expression, which was not included in our previous publication [5], and found no statistically significant change (*p* = 0.687).

### 3.3. E-CADHERIN Expression in Eutopic Endometrium

**Figure 1:**
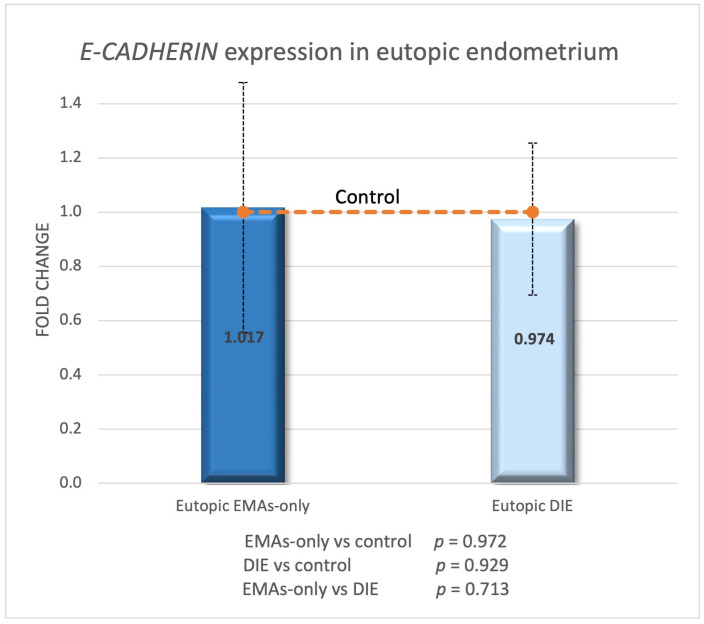
*E-CADHERIN* expression in eutopic endometrium of EMAs-only and DIE groups compared to normal endometrium.

*E-CADHERIN* expression was measured in normal endometrium (control group, *n* = 8) and compared to eutopic endometrium from EMAs-only (*n* = 9) and DIE groups (*n* = 10). There was no change in eutopic endometrium between the EMAs-only and DIE groups compared to normal endometrium (Figure 1).

### 3.4. E-CADHERIN Expression in Paired Endometrial Tissues

**Figure 2:**
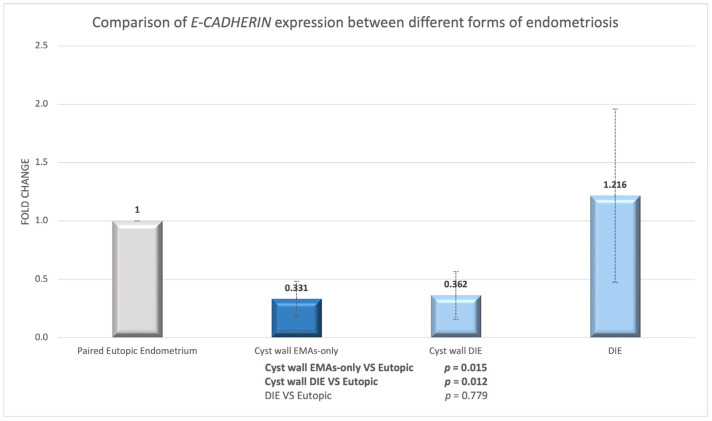
*E-CADHERIN* expression in endometriotic cyst wall or nodule compared to their paired eutopic endometrium.

We then examined the mRNA expression of *E-CADHERIN* in women with endometriosis, namely in eutopic endometrium and paired endometriotic lesions. In women with EMAs-only, *E-CADHERIN* expression was nearly three times lower in the endometriotic cyst walls than in the matched eutopic endometrium (*n* = 9, *p* = 0.015; Figure 2).

In women with DIE, the endometriotic cyst wall had the same substantial *E-CADHERIN* under-expression compared to eutopic endometrium, but there was no significant difference in expression between eutopic endometrium and DIE lesions (eutopic-cyst *p* = 0.012; eutopic-DIE *p* = 0.779; Figure 2). We discovered no significant difference between the endometriotic cyst wall and the DIE lesion (cyst-DIE *p* = 0.201). However, there was a fourfold increase in expression in DIE lesions compared to endometriotic cysts.

### 3.5. E-CADHERIN Expression in Endometriotic Cysts Between Women with and Without DIE

**Figure 3:**
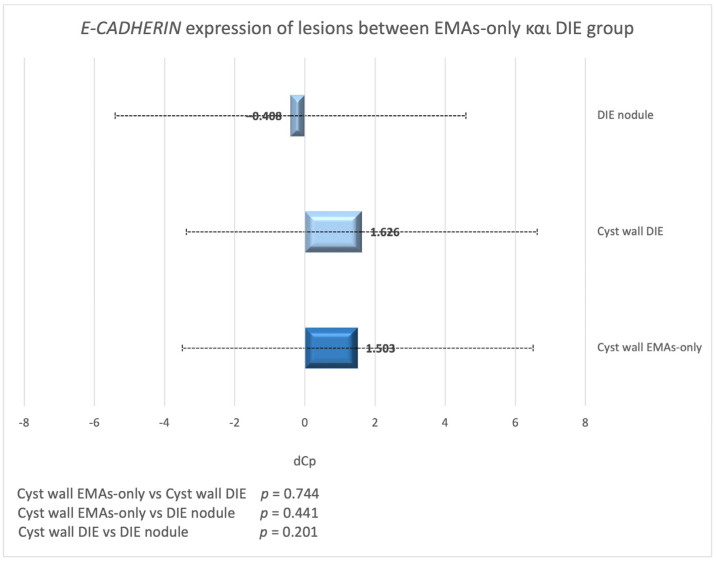
dCp value of *E-CADHERIN* in endometriotic cysts and nodules in DIE and EMAs-only group.

No significant difference in *E-CADHERIN* expression was found between endometriotic cysts of EMAs-only and the DIE group (*p* = 0.744; Figure 3). The same result was observed between endometriotic cysts of the EMAs-only group and DIE lesions (*p* = 0.441; Figure 3).

### 3.6. miR-200b Expression in Eutopic Endometrium

**Figure 4:**
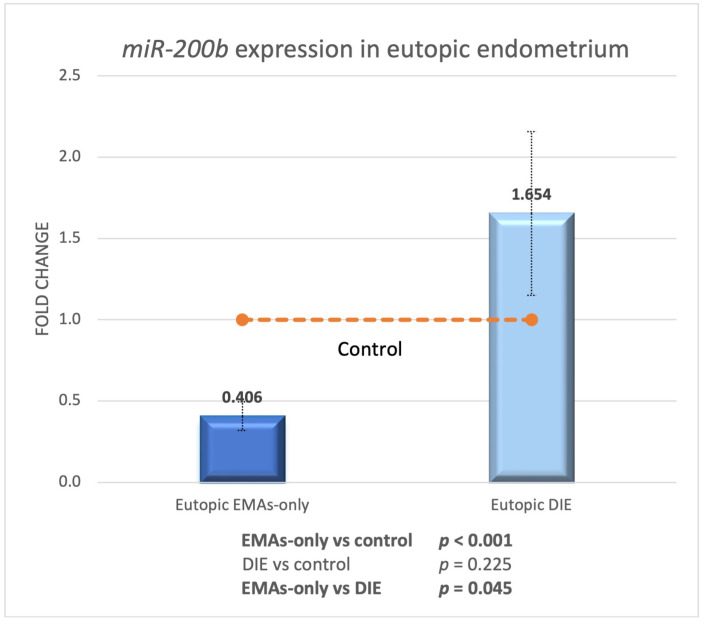
*miR-200b* expression in eutopic endometrium of EMAs-only and DIE groups compared to normal endometrium.

We examined the expression levels of *miR-200b* in the eutopic endometrium of control, EMAs-only, and DIE groups. In EMAs-only endometrium, *miR-200b* expression was 2.46 times lower than in normal endometrium, whereas there was no change in the DIE group (EMAs-only-control *p* ≤ 0.001, DIE-control *p* = 0.225; Figure 4). When we compared the expression of DIE to the EMAs-only group, we discovered that *miR-200b* was substantially over-expressed fourfold in the DIE group (EMAs-only-DIE *p* = 0.045).

### 3.7. miR-200b Expression in Paired Endometrial Tissues

**Figure 5:**
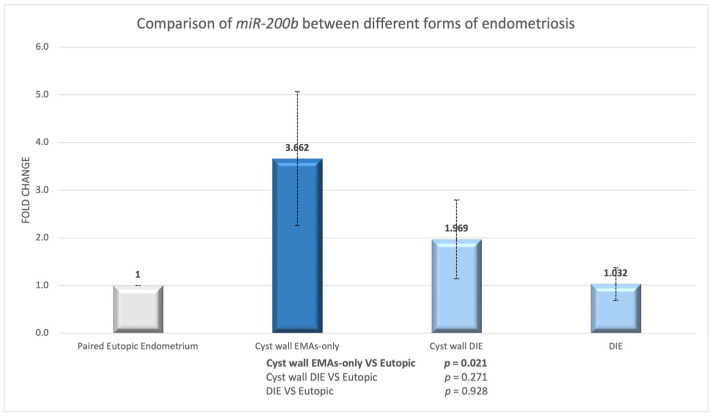
*miR-200b* expression in endometriotic cyst wall or nodule compared to their paired eutopic endometrium.

The expression of *miR-200b* was also examined in matched eutopic endometrium and endometriosis lesions. In the EMAs-only group, *miR-200b* was significantly over-expressed in endometriotic cyst walls compared to matched eutopic endometrium (*p* = 0.021; Figure 5).

In women with DIE, there was no significant difference in expression between endometriotic cyst wall and DIE lesion compared to eutopic endometrium (eutopic-cyst *p* = 0.271; eutopic-DIE *p* = 0.928; Figure 5). However, there was an approximately 2-fold increase in expression in the endometriotic cyst wall compared to the eutopic endometrium. We discovered no significant difference between the endometriotic cyst wall and the DIE lesion (cyst-DIE *p* = 0.453). However, there was a similar tendency of greater expression, nearly twofold, in the endometriotic cyst compared to the DIE lesion.

### 3.8. miR-200b Expression in Endometriotic Cysts Between Women with and Without DIE

**Figure 6:**
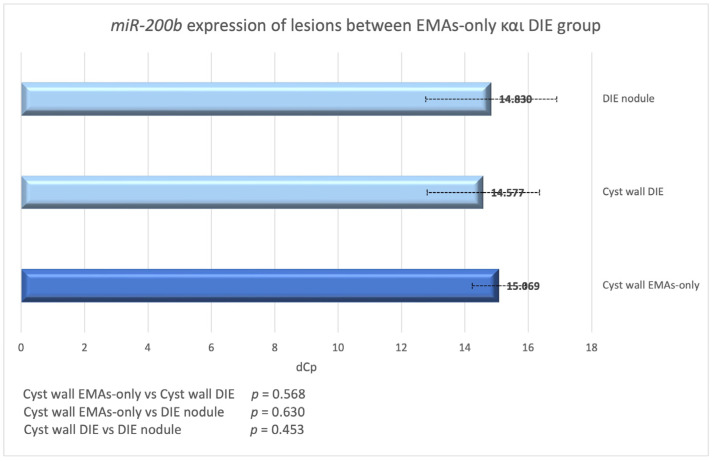
dCp value of *miR-200b* in endometriotic cysts and nodules in DIE and EMAs-only group.

When we compared the endometriotic cysts of the EMAs-only and DIE group, we found no significant differences in *miR-200b* expression (*p* = 0.568; Figure 6). Additionally, no significant difference was found when we compared the endometriotic cysts of the EMAs-only group with the DIE lesions (*p* = 0.630).

## 4. Discussion

Epithelial–Mesenchymal Transition is a multi-stage process in which epithelial cells modify their cytoskeleton, apical-basal polarity and cell-to-cell interactions in order to acquire a mesenchymal cell phenotype and express mesenchymal markers [8,9]. EMT’s most significant effect on an epithelial cell is the downregulation of *E-CADHERIN* expression, which is regulated by multiple mesenchymal transcription factors such as *SNAIL*, *SLUG*, *TWIST* and *ZEB1*. EMT is thought to have a role in endometriosis pathogenesis beginning with the implantation of endometriotic cells and continuing through the early stages of lesion growth. EMT in endometriosis causes the formation of intermediate cell states with hybrid epithelial/mesenchymal phenotypes [10]. Konrad et al. proposed that in endometriosis, EMT is only partial, initiated by the ectopic microenvironment and activated after endometriotic cell implantation but without loss of epithelial phenotype [10]. As a result, we may conclude that the epigenetic/genetic abnormalities cause the endometrial epithelial cell to adopt a more mesenchymal phenotype that bears even in the eutopic endometrium when the cell is exposed to a different micro-environment with increased hypoxia or/and high estradiol concentrations that trigger further epigenetic/genetic changes [2,11].

*E-CADHERIN*’s significance in endometriosis is less apparent and important than it is in carcinogenesis. Several studies on the expression of the *E-CADHERIN* gene in women with endometriosis yielded inconsistent results. Matsuzaki et al. discovered that epithelial cells in DIE lesions exhibited greater *E-CADHERIN* expression than endometriomas and menstrual endometrium [12]. Biyik et al. discovered that DIE lesions had the lowest expression of the *E-CADHERIN* gene compared to endometriomas and normal endometrium [13]. However, the most significant finding was the presence of epithelial cells in endometriotic lesions that did not express *E-CADHERIN*, indicating that these cells induce EMT and infiltrate [14,15,16]. This finding suggests that in DIE lesions, the infiltrating nature may be related to certain cell populations that have lost *E-CADHERIN* expression and completed the change to a mesenchymal phenotype. Loss of *E-CADHERIN* expression in endometrial epithelial cells can activate the Wnt/β-catenin pathway, leading to increased cell motility and invasion, as demonstrated by in vitro experiments [17]. Furthermore, the Wnt/β-catenin pathway inhibits *ZEB1* expression, a regulator of *E-CADHERIN*, perhaps through *miR-33b* interference [18]. This suggests that the Wnt/β-catenin system regulates the *ZEB1-E-CADHERIN* axis via miR gene-mediated transcription.

Several research has documented the expression of *miR-200b* in endometriosis, however, it has not been thoroughly examined. The key finding is that *miR-200b* expression is reduced in endometriotic lesions compared to eutopic endometrium in both women with and without endometriosis [19,20,21,22]. Furthermore, *miR-200b* expression was higher in mesenchymal stem cells of the menstrual endometrium and stromatic cells in women with endometriosis than in those without [23,24,25].

Although the gene expression profile of lesions can provide information about the genes that play critical roles in disease pathogenesis, the most significant understanding is how these genes interact with one another at the molecular pathway level, influencing the cellular phenotype. It is well established that *miR-200b* and *ZEB1* are regulated in a double-negative feedback loop fashion, with overexpression of *miR-200b* downregulating *ZEB1* and promoting the epithelial phenotype and inhibition of *miR-200b* promoting EMT and the mesenchymal phenotype in cell cultures [6,26]. Furthermore, inhibition and overexpression of *miR-200b* resulted in reversible mesenchymal and epithelial phenotypes [6]. This suggests that miR-200b-ZEB1 double-negative feedback loop regulation is reversible, even if a particular phenotype is identified.

To our knowledge, no work has concurrently explored the *miR-200b-ZEB1* feedback loop and *E-CADHERIN* expression in various kinds of endometriosis. However, studies have looked at two of these genes in the same samples. Furuya et al. discovered that epithelial cells in DIE lesions express more *ZEB1* than in endometriomas, but both lesions express *E-CADHERIN* [14]. Wu et al. discovered that eutopic endometrium had higher *ZEB1* expression than endometriomas, but eutopic and ectopic endometrium of endometriosis had lower *E-CADHERIN* expression than normal endometrium [27]. Esfandriari et al. discovered higher expression of *ZEB1* in ectopic endometrium, but the same levels of *E-CADHERIN* expression between normal and ectopic endometrium [28]. Logan et al. found increased expression of *ZEB1* in epithelial cells of eutopic endometrium in women with endometriosis and decreased expression in stromatic cells, while the *miR-200b* showed increased expression in stromatic cells [25]. Overexpression of *miR-200b* decreased the *ZEB1* levels and increased the *E-CADHERIN* expression in endometriotic cell lines [29].

As a follow-up to our previous investigation, in which we discovered a differential expression profile of *ZEB1* in various endometriotic lesions [5], we evaluated the expression of *E-CADHERIN* and *miR-200b* in various kinds of endometriosis. These three genes interact with one another and have key roles in EMT, particularly carcinogenesis [30]. Although we recognize that EMT and endometriosis pathogenesis are complex and multi-step processes influenced by numerous molecular pathways, we attempted to investigate the regulation of the most important epithelial marker, *E-CADHERIN*, in various endometriotic lesions, as well as how *E-CADHERIN* expression is regulated by other genes.

First, we compared the expression of genes in eutopic endometrium between women with and without endometriosis. In this comparison, the endometriosis group included women with ovarian, deep infiltrative, or both types of lesions. The study found no significant difference in gene expression between eutopic and normal endometriums for *miR-200b*, *ZEB1*, or *E-CADHERIN* (*ΖΕB1 p* = 0.687, *E-CADHERIN p* = 0.982, *miR-200b p* = 0.836). However, when women with endometriosis were divided into subgroups based on the kind of endometriosis, we discovered differences in gene expression patterns between the two endometriosis groups and the normal endometrium. As a result, our first conclusion is that various types of endometriosis display diverse gene expression patterns in the eutopic endometrium and should not be classified as a common study group.

Combining the findings from our past and present studies, we discovered that *ZEB1* and *miR-200b* had comparable expression patterns in the eutopic endometrium of women with and without deep infiltrative endometriosis when compared to normal endometrium. *ZEB1* and *miR-200b* are significantly under-expressed in the eutopic endometrium of women without deep infiltrative endometriosis (*ZEB1 p* = 0.001, *miR-200b p* < 0.001), while they are over-expressed in the eutopic endometrium of women with deep infiltrative endometriosis (*ZEB1 p* = 0.207, *miR-200b p* = 0.225). *E-CADHERIN* expression levels are similar in the eutopic endometrium of the two endometriosis groups and the normal endometrium.

When we analyzed the expression profiles of these genes across the two endometriosis groups, we discovered that the eutopic endometrium of deep infiltrative endometriosis exhibits considerable overexpression of *ZEB1* (*p* = 0.014) and *miR-200b* (*p* = 0.045). However, *E-CADHERIN* expression was similar in both endometriosis groups (*p* = 0.713). This indicates that the epithelial phenotype is preserved in the eutopic endometrium of women with and without profound infiltrative endometriosis. However, the expression of mesenchymal genes (*ZEB1*) and EMT suppressor genes (*miR-200b*) differs across the two endometriosis groups. In the eutopic endometrium of women with deep infiltrative endometriosis, the balance is maintained by the over-expression of both activating and suppressive genes of the mesenchymal phenotype, while in the eutopic endometrium of women with simple ovarian endometriosis the balance is maintained by under-expression of such genes.

In the endometriotic cysts, we found a decrease in *E-CADHERIN* expression in both endometriosis groups, indicating that the mesenchymal phenotype is accentuated in these lesions. However, we discovered that *ZEB1* and *miR-200b* expression were differentially regulated between the two endometriosis subgroups, ranging from eutopic endometrium to endometriotic cysts.

**Figure 7:**
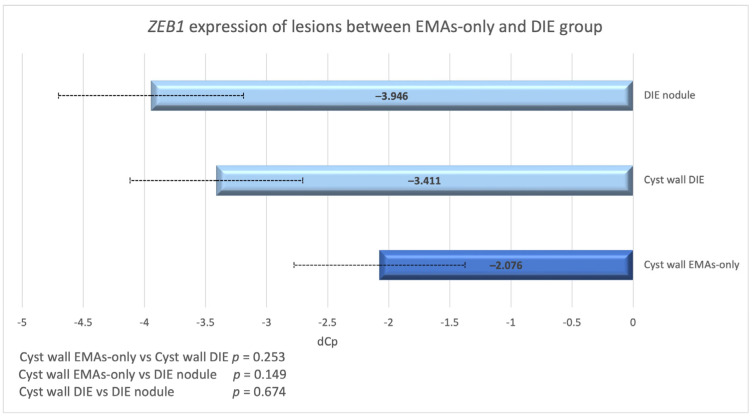
dCp value of *ZEB1* in endometriotic cysts and nodules in DIE and EMAs-only group. There is a gradual increase in *ZEB1* expression from the cyst wall of EMAs-only to the cyst wall of DIE and finally to the DIE nodule.

*ZEB1* expression was reduced in cysts from the deep infiltrative endometriosis group, but raised in simple ovarian endometriotic cysts when compared to their associated eutopic endometrium. *MiR-200b* expression was increased in endometriotic cysts from both deep infiltrative and ovarian endometriosis. However, when endometriotic cysts from the two subgroups were compared in terms of *ZEB1* expression, it was shown that in the DIE sub-group expression of this gene was still 2.5 times higher compared to that found in the cysts of the non-DIE group. This difference though was not statistically significant (*p* = 0.253; Figure 7).

Combining these findings, we may infer that implantation of an ectopic endometrial cell in women with deep infiltrative endometriosis in the ovary is linked with lower *ZEB1* expression and higher *miR-200b* expression when compared to the matched eutopic endometrium. Despite the decline in *ZEB1* expression, the cell will still develop a more mesenchymal phenotype via lowering *E-CADHERIN* expression. This cell appears to have already been genetically/epigenetically predisposed to take this path toward EMT. In contrast, in women with uncomplicated ovarian endometriosis, implanting endometrial cells in the ovary increases both *ZEB1* and *miR-200b* expression while decreasing *E-CADHERIN* expression. The cells in this endometriosis sub-group will require a higher molecular boost to acquire the same EMT stage.

These discrepancies suggest that endometrial cells in the deep infiltrative and simple ovarian endometriosis subgroups use distinct regulatory mechanisms to reduce *E-CADHERIN* expression when they implant in the ovary. The expression profile of these genes differs significantly between endometriotic cysts of deep infiltrative and ovarian endometriosis, with *ZEB1* showing a tendency of higher expression in the deep infiltrative endometriosis group, implying that there is an enhanced mesenchymal dynamic in these cysts, derived from an already primed eutopic endometrium. This conclusion is consistent with the notion that endometriotic cysts in individuals with deep infiltrative endometriosis are usually more difficult to dissect, have greater fibrosis and vascularity of the surrounding ovarian stroma, and have surgical planes that are more difficult to recognize and follow [31,32,33].

As a result, endometrial cells from various types of endometriosis have varied gene expression patterns and will follow distinct molecular pathways to acquire a partial EMT state with a more mesenchymal phenotype, eventually developing into endometriotic ovarian cystic tumors.

The DIE nodule, on the other hand, has a comparable expression profile to the paired eutopic endometrium for the genes *ZEB1* (*p* = 0.967), *miR-200b* (*p* = 0.928), and *E-CADHERIN* (*p* = 0.779). These results are consistent with the discovery that deep infiltrating implants at a depth of more than 5 mm exhibit comparable cyclicity of the menstrual cycle and are exposed to similar hormone levels as the eutopic endometrium [34]. As a result, we may hypothesize that when ectopic endometrial cells implant on the peritoneum, they acquire additional genetic/epigenetic abnormalities and will be able to infiltrate the peritoneum to a depth that the local environmental conditions resemble those of the endometrial cavity. In such depth, they will develop slowly, in order to create the fibromuscular capsule that characterizes the lesion and infiltrate adjacent tissues and organs.

## 5. Conclusions

In the eutopic endometrium of women with endometriosis, there is a balanced expression of mesenchymal genes (*ZEB1*) and their suppressors (*miR-200b*), which maintains the epithelial phenotype of the cells. The eutopic endometrium of deep infiltrative endometriosis is characterized by a state of *ZEB1* and *miR-200b* over-expression while at ovarian endometriosis there is a state of under-expression of these genes in the eutopic endometrium. In both forms of endometriosis, the epithelial phenotype is maintained but with two opposing balanced states of gene expression.

In the endometriotic cysts, the epithelial phenotype weakens while the mesenchymal is enhanced, at the same level for both forms of endometriosis but with different molecular pathways. The hyper-estrogenic environment of the ovary results in decreased *E-CADHERIN* expression, enhancing the mesenchymal phenotype. The investigation of *ZEB1* and *miR-200b* expression shows that the epithelial or mesenchymal phenotype of the endometriotic cell is regulated by the change in a dynamic balance between the expression of genes that promote the epithelial or mesenchymal state under the effect of the ovarian environment.

The DIE nodule is a lesion with increased mesenchymal dynamic. When the implant is exposed to the hostile environment of the peritoneal cavity, it acquires additional genetic/epigenetic defects that enable the implant to infiltrate to a depth where the environment resembles the endometrial cavity.

## Data Availability

The data are given in the paper.
